# Marine Compound 3-Bromo-4,5-dihydroxybenzaldehyde Protects Skin Cells against Oxidative Damage via the Nrf2/HO-1 Pathway

**DOI:** 10.3390/md17040234

**Published:** 2019-04-19

**Authors:** Yea Seong Ryu, Pincha Devage Sameera Madushan Fernando, Kyoung Ah Kang, Mei Jing Piao, Ao Xuan Zhen, Hee Kyoung Kang, Young Sang Koh, Jin Won Hyun

**Affiliations:** School of Medicine and Jeju Research Center for Natural Medicine, Jeju National University, Jeju 63243, Korea; rmj5924@naver.com (Y.S.R.); sameeramadhu91@gmail.com (P.D.S.M.F.); legna07@naver.com (K.A.K.); meijing0219@hotmail.com (M.J.P.); zhenaoxuan705@gmail.com (A.X.Z.); pharmkhk@jejunu.ac.kr (H.K.K.); yskoh7@jejunu.ac.kr (Y.S.K.)

**Keywords:** keratinocytes, 3-bromo-4,5-dihydroxybenzaldehyde, heme oxygenase-1, nuclear factor erythroid 2-related factor 2, cytoprotection

## Abstract

In this study, we aimed to illustrate the potential bio-effects of 3-bromo-4,5-dihydroxybenzaldehyde (3-BDB) on the antioxidant/cytoprotective enzyme heme oxygenase-1 (HO-1) in keratinocytes. The antioxidant effects of 3-BDB were examined via reverse transcription PCR, Western blotting, HO-1 activity assay, and immunocytochemistry. Chromatin immunoprecipitation analysis was performed to test for nuclear factor erythroid 2-related factor 2 (Nrf2) binding to the antioxidant response element of the HO-1 promoter. Furthermore, the 3-(4,5-dimethylthiazol-2-yl)-2,5-diphenyltetrazolium bromide assay showed that the cytoprotective effects of 3-BDB were mediated by the activation of extracellular signal-regulated kinase (ERK) and protein kinase B (PKB, Akt) signaling. Moreover, 3-BDB induced the phosphorylation of ERK and Akt, while inhibitors of ERK and Akt abrogated the 3-BDB-enhanced levels of HO-1 and Nrf2. Finally, 3-BDB protected cells from H_2_O_2_- and UVB-induced oxidative damage. This 3-BDB-mediated cytoprotection was suppressed by inhibitors of HO-1, ERK, and Akt. The present results indicate that 3-BDB activated Nrf2 signaling cascades in keratinocytes, which was mediated by ERK and Akt, upregulated HO-1, and induced cytoprotective effects against oxidative stress.

## 1. Introduction

The balance between reactive oxygen species (ROS) production and eradication of their toxicity is crucial to maintaining the skin redox balance. The skin is one of the most important targets of oxidative stress due to ROS from exposure to environmental stimuli and endogenous reactions [1,2]. ROS are a group of free radicals which affect macromolecules (DNA, lipids, and proteins), resulting in the generation of other reactive species [3,4]. ROS are generated during normal metabolism and rapidly induce antioxidant enzymes to maintain cellular homeostasis. The skin possesses an antioxidant defense system; however, it can be disrupted by excessive ROS, leading to oxidative damage, atopic dermatitis, premature skin aging, and skin cancer [5]. As a cytoprotective signaling mechanism, the Kelch-like ECH-associated protein 1 (Keap1)-nuclear factor erythroid 2-related factor 2 (Nrf2) pathway can help prevent and treat oxidative damage and related diseases [6]. 

Under normal conditions, Nrf2 has a short half-life and is continuously subjected to ubiquitination and degradation via the sequestration of the negative regulator Keap1 [7]. However, under conditions of oxidative stress, Nrf2 ubiquitination and degradation are blocked, thus allowing for Nrf2 phosphorylation and nuclear translocation [8]. In the nucleus, Nrf2 binds to the antioxidant response element (ARE) in the promoter region of target genes, including heme oxygenase-1 (HO-1), thereby inducing their expression [9]. 

HO-1 is an antioxidant enzyme that is induced as a response to cellular oxidative stress, and it regulates the rate-limiting step of heme catabolism, resulting in the production of carbon monoxide, ferrous iron, and biliverdin. Biliverdin is further degraded to bilirubin by the action of biliverdin reductase [10]. Intracellular ROS are neutralized by the end products of heme catabolism, indicating the antioxidant properties of these products [11]. HO-1 gene expression has been shown to be regulated at the transcriptional level and enhanced by Nrf2 nuclear translocation [12,13,14], resulting from the activation of various signal transduction pathways, including extracellular signal-regulated kinase (ERK) and protein kinase B (PKB, Akt). HO-1 pla ys an important role in cytoprotection against oxidative damage [15,16,17]. In addition, HO-1 provides cytoprotection against ROS in the skin [18,19]. 

The natural antioxidant compound 3-bromo-4,5-dihydroxybenzaldehyde (3-BDB) has been isolated from marine red algae such as *Rhodomela confervoides*, *Polysiphonia morrowii*, and *Polysiphonia urceolata* [20,21,22]. The 3-BDB compound exerts radical scavenging and antiviral effects against different types of viruses such as infectious pancreatic necrosis virus and fish pathogenic infectious hematopoietic necrosis virus [21,22], while also exerting photoprotective effects on human keratinocytes that are exposed to ultraviolet B-mediated oxidative stress [23]. However, the mechanisms underlying the cytoprotective effects of 3-BDB against oxidative stress are unclear. 

In this study, we investigated the mechanisms underlying 3-BDB-mediated cytoprotection against oxidative stress in human keratinocytes, with a primary focus on the stimulatory effects of 3-BDB on HO-1 activity. Furthermore, we analyzed the roles of the ERK- and Akt-Nrf2 signaling cascades in this cytoprotection and HO-1 induction.

## 2. Results

### 2.1. HO-1 Activity and Expression Are Induced by 3-BDB in a Concentration- and Time-Dependent Manner

**Figure 1:**
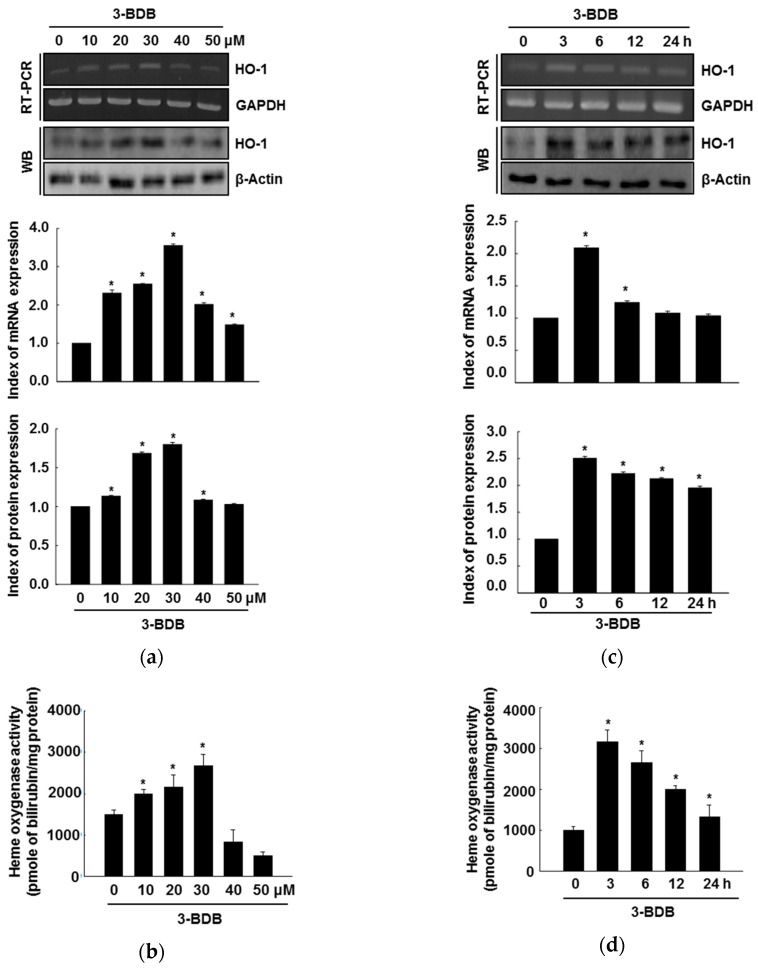
Effects of 3-bromo-4,5-dihydroxybenzaldehyde (3-BDB) on the levels and bioactivity of heme oxygenase-1 (HO-1) in a concentration- and time-dependent manner. HaCaT cells were pretreated with 3-BDB and incubated for 24 h with the indicated concentrations. (**a**,**c**) RT-PCR and Western blotting (WB) were used to analyze the HO-1 mRNA and protein expression. Glyceraldehyde-3-phosphate dehydrogenase (GAPDH) was used as the loading control. (**b**,**d**) The amount of bilirubin formed was used as an indicator, to assess HO-1 activity. * Significant difference compared to the control (*p* < 0.05).

HaCaT cells were pretreated with 3-BDB at selected concentrations of 10 µM, 20 µM, 30 µM, 40 µM and 50 µM to determine its effects on HO-1 expression. HO-1 mRNA and protein expression levels were enhanced upon treatment with 10 µM 3-BDB and they were further increased with treatment up to 30 µM 3-BDB compared with the levels in the untreated control cells (Figure 1a). However, 40 µM and 50 µM 3-BDB downregulated HO-1 mRNA and protein expression relative to the expression with 30 µM 3-BDB. It has been reported that 3-BDB is not cytotoxic at lower concentrations (10 µM, 20 µM, and 30 µM). However, 3-BDB was shown to be cytotoxic at higher concentrations (40 µM and 50 µM) [24]. Consistent with the mRNA and protein levels, HO-1 activity was upregulated upon 3-BDB treatment (Figure 1b), exhibiting a peak at 30 µM. According to these results, we decided to use 30 µM 3-BDB as the optimum concentration for subsequent experiments.

To examine the time-dependent effects of 3-BDB, HaCaT cells were pretreated with 30 µM 3-BDB and HO-1 expression was observed for 24 h. HO-1 mRNA and protein was upregulated within three hours following 3-BDB treatment (Figure 1c). This temporal upregulation was closely associated with an increase in HO-1 activity over time (Figure 1d). These results suggest that 3-BDB has a biological antioxidant effect in HaCaT cells.

### 2.2. Protein Expression, Nuclear Translocation, and ARE Binding of Nrf2 Are Enhanced by 3-BDB

**Figure 2:**
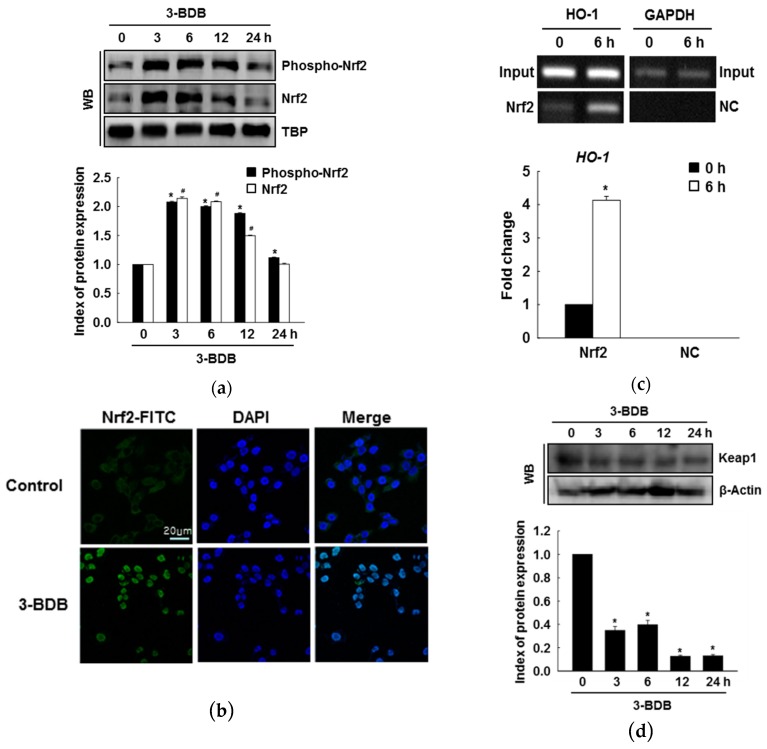
Effect of 3-BDB (30 µM) on the protein expression, nuclear translocation, and Nrf2 binding to antioxidant response element. **(a**) The expressions of phospho-Nrf2 and Nrf2 were detected by Western blotting of nuclear extracts. (**b**) Cells with Nrf2 are shown in green; the nuclei are shown in blue. Merged images show the nuclear translocation of Nrf2. Green represented Nrf2-fluorescein isothiocyanate (FITC) stained cells and blue represented 4′,6-diamidino-2-phenylindole (DAPI) stained nucleus. (**c**) Binding of Nrf2 to the ARE sequence in the HO-1 promoter was analyzed via chromatin immunoprecipitation (ChIP) followed by PCR. NC: negative control. (**d**) Keap1 expression was analyzed via Western blotting. * Significant difference compared to the control (*p* < 0.05).

Several cytoprotective enzymes are regulated by the transcription factor Nrf2. HO-1 is also regulated by Nrf2. Accordingly, we examined whether 3-BDB stimulates phosphorylation and nuclear translocation of Nrf2. Treatment with 3-BDB upregulated Nrf2 expression and increased Nrf2 phosphorylation, indicating that 3-BDB temporally increased the nuclear accumulation of Nrf2 (Figure 2a). Nrf2 nuclear translocation induced by 3-BDB was further supported by immunocytochemical analysis (Figure 2b). Furthermore, when untreated cells were compared with 3-BDB treated cells, a significant enhancement in Nrf2 binding to the ARE in the HO-1 promoter region was observed in the treated cells, which was assessed with a chromatin immunoprecipitation (ChIP) assay (Figure 2c). Keap1 was downregulated after treatment with 3-BDB in a time-dependent manner (Figure 2d).

### 2.3. The Compound 3-BDB Induces HO-1 by Mediating Nrf2

**Figure 3:**
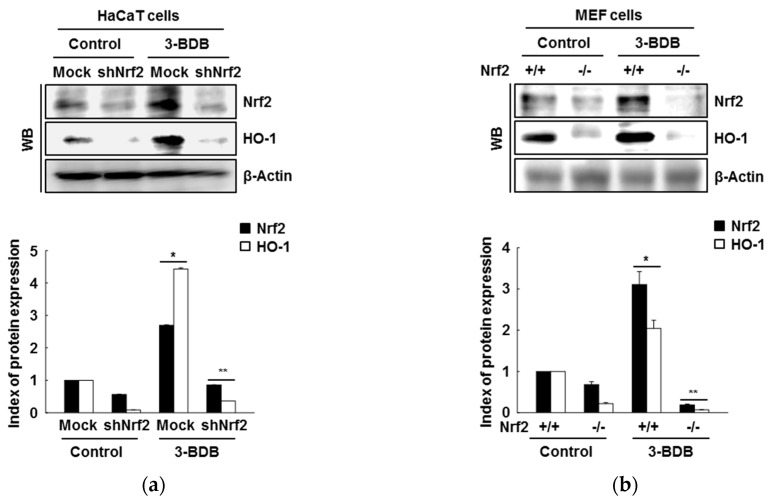
Role of Nrf2 in the 3-BDB (30 µM)-mediated enhancement of Nrf2 and HO-1 expression. (**a**) HaCaT cells were transfected with shRNA-control and shRNA-Nrf2 plasmids and treated with 3-BDB. Western blotting was used to analyze the proteins in the whole cell lysates. (**b**) Nrf2-WT or Nrf2-deficient mouse embryonic fibroblast (MEF) cells were treated 3-BDB, and Western blotting was used to measure the levels of HO-1 and Nrf2. * Significant difference compared with control (*p* < 0.05); ** significant difference compared with 3-BDB-treated cells (*p* < 0.05).

To investigate whether target gene transcription is upregulated by Nrf2, we evaluated Nrf2 and HO-1 expression after short hairpin RNA (shRNA)-mediated Nrf2 knockdown. Nrf2 silencing abrogated 3-BDB-induced HO-1 expression (Figure 3a). Moreover, treatment of mouse embryonic fibroblasts (MEFs) derived from Nrf2-deficient mice with 3-BDB attenuated the increase in Nrf2 and HO-1 levels (Figure 3b).

### 2.4. The Compound 3-BDB Activates Expression of HO-1 and Nrf2 via Phosphorylation of ERK and Akt

**Figure 4:**
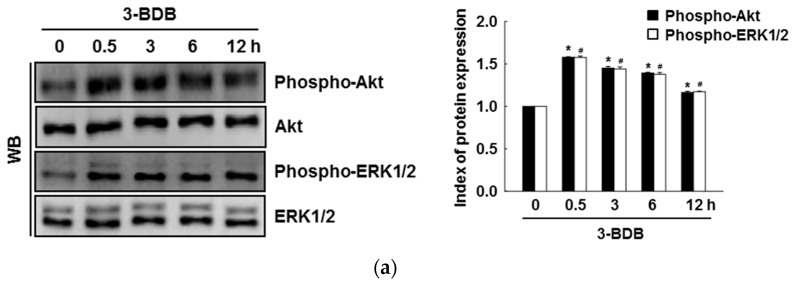

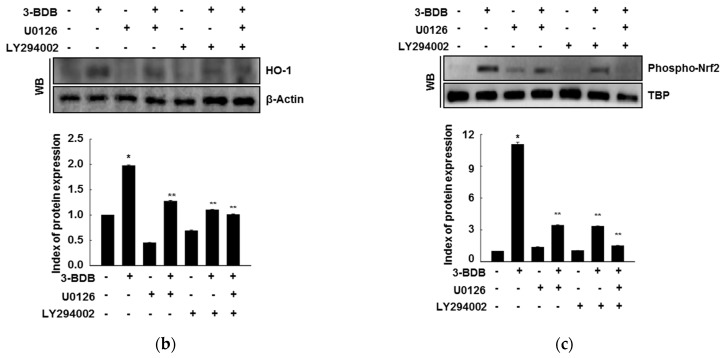
The extracellular signal-regulated kinase (ERK) and the protein kinase B (Akt) signaling pathways are affected by 3-BDB (30 µM) treatment. (**a**) After treatment with 3-BDB, total protein lysates were collected and analyzed for the levels of phospho-Akt, Akt, phospho-ERK1/2, and ERK1/2 with Western blotting using specific antibodies. (**b**) HO-1 and (**c**) phospho-Nrf2 levels were analyzed via Western blotting. U0126 and LY294002 are an ERK inhibitor and a PI3K inhibitor, respectively. * Significant difference compared with control (*p* < 0.05); ** significant difference compared with 3-BDB-treated cells (*p* < 0.05).

Treatment with 3-BDB (30 µM) significantly enhanced the phosphorylation of ERK and Akt in a time-dependent manner within 30 min of treatment (Figure 4a). To further examine whether ERK and Akt activation are caused by the cytoprotective effects of 3-BDB, cells were pretreated with U0126 and LY294002 (ERK inhibitor and Akt inhibitor, respectively) one hour before treatment with 3-BDB. U0126 and LY294002 notably attenuated HO-1 expression and Nrf2 phosphorylation upon 3-BDB treatment (Figure 4b,c). These results suggest that the protective effect of 3-BDB is mediated by ERK and Akt signaling, an important upstream signaling pathway that regulates Nrf2/HO-1.

### 2.5. Cytoprotective Effects of 3-BDB Are Mediated by Activation of ERK and Akt Signaling

**Figure 5:**
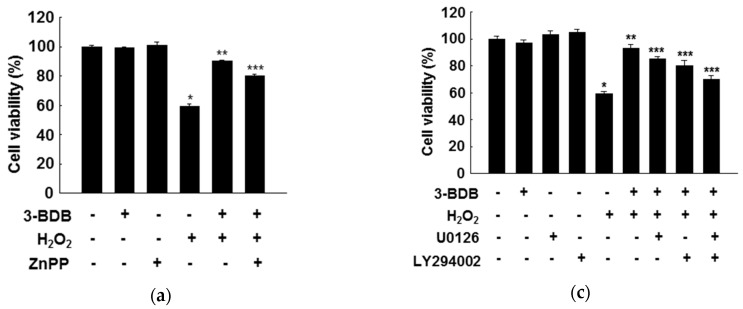

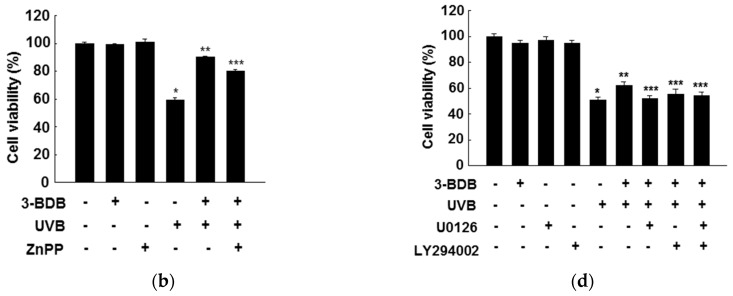
Cytoprotective effect of 3-BDB (30 µM) against cellular oxidative stress. HaCaT cells were pretreated with zinc protoporphyrin (ZnPP), along with 3-BDB, and exposed to (**a**) H_2_O_2_ or (**b**) UVB radiation. Cells were pretreated with U0126 or LY294002 and/or 3-BDB and exposed to (**c**) H_2_O_2_ or (**d**) UVB radiation. Cell viability was analyzed after 24 h by the 3-(4,5-dimethylthiazol-2-yl)-2,5-diphenyltetrazolium bromide (MTT) assay. * Significant difference compared with control (*p* < 0.05); ** significant difference compared with H_2_O_2_- or UVB-treated cells (*p* < 0.05); *** significant difference compared with 3-BDB + H_2_O_2_- or 3-BDB + UVB -treated cells (*p* < 0.05).

To determine whether the cytoprotective effect of 3-BDB is related to the upregulation of HO-1 activity, HaCaT cells were pretreated with zinc protoporphyrin (ZnPP), a HO-1 inhibitor. ZnPP markedly inhibited the protective effect of 3-BDB against H_2_O_2_- or UVB-induced cytotoxicity, suggesting that HO-1 induction is responsible for the enhancement of cell viability in 3-BDB-pretreated cells under oxidative stress (Figure 5a,b). Thus, the cytoprotection by 3-BDB probably involves HO-1. 

Furthermore, we examined whether ERK and PI3K/Akt signaling regulates HO-1 and Nrf2 expression in response to oxidative stress. The protection of 3-BDB against H_2_O_2_ and UVB exposure was suppressed by U0126 and LY294002. (Figure 5c,d). We hypothesize that the protective effect of 3-BDB is regulated by ERK and Akt signaling pathways, which induce HO-1 and Nrf2 expression.

## 3. Discussion

The epidermis and its associated cells are equipped with essential protective mechanisms, especially antioxidant systems that protect from oxidative stress. A previous study reported that 3-BDB has the ability to ameliorate redox reaction-mediated damage, especially within cells and tissues. In addition, 3-BDB was suggested to be a powerful antioxidant in human keratinocytes [25] and it exhibited antiviral activity against hematopoietic necrosis virus [21]. It has also been reported that 3-BDB can reduce intracellular ROS generation through harmful stimuli (hydrogen peroxide and UVB radiation), superoxide anion generation (xanthine/xanthine oxidase system), and hydroxyl radical generation (Fenton reaction). It has been specifically mentioned that 3-BDB can protect human keratinocytes from UVB-induced oxidative stress [23]. Keratinocytes are most frequently exposed to endogenous and exogenous pro-oxidant agents with deleterious effects on the skin.

HO-1, an inducible cytoprotective enzyme involved in cellular defense, plays a crucial role in adaptation to oxidative stress in different cell types. Several studies have reported that upregulated HO-1 was involved in the cellular defense system as a response to oxidative stress [26]. The present study shows that 3-BDB significantly upregulates levels of HO-1 mRNA and protein and HO-1 activity via the ARE sequence in the promoter region of HO-1. Furthermore, the inhibition of HO-1 eradicated the protective effect of 3-BDB against oxidative stress-mediated cellular damage. These data indicate that HO-1 upregulation in keratinocytes is responsible for the 3-BDB-mediated protective effect against oxidative stress.

Several compounds extracted from natural products have an ability to induce Nrf2-mediated HO-1 levels through the activation of Akt and ERK. For example, furotrilliumoside isolated from *Trillium tschonoskii* Maxim, sophoraflavonone G from *Sophora alopecuroides*, and morin isolated from Chinese herbs suppress oxidative stress by regulating the mitogen-activated protein kinase (MAPK), Akt, and Nrf2/HO-1 signaling pathways [27,28,29]. We previously reported that 7,8-dihydroxyflavone and eckol induce the Nrf2-mediated antioxidant enzyme HO-1 by activating ERK and Akt signaling pathways [26,30]. The present results indicate that 3-BDB has particularly potent protective effects. 

Nrf2 modulates expression of phase II enzymes and is considered an upstream transcription factor [31]. Under normal conditions, Nrf2 is sequestered in the cytoplasm through its binding to Keap1. Oxidative and electrophilic stress provokes physiological responses, which leads to dissociation of Nrf2 from its docking protein and translocation to the nucleus [7,8]. The nuclear translocation of Nrf2 upregulates antioxidant genes, thus playing a crucial role in the induction of HO-1; it may provide an important therapeutic strategy against oxidative stress-mediated cell damage [32]. In the present study, 3-BDB treatment significantly upregulated Nrf2 expression and nuclear translocation, and it downregulated Keap1. However, because of the short half-life of Nrf2, it was downregulated in response to 3-BDB at 12 h. These results show that 3-BDB may retard Keap1-dependent Nrf2 degradation. Furthermore, the present results indicate that upregulation of HO-1 expression is regulated by Nrf2 activation in keratinocytes. Similarly, docosahexaenoic acid increases Keap1 degradation and prevents Keap1-mediated inactivation of Nrf2 [33]. 

**Figure 6:**
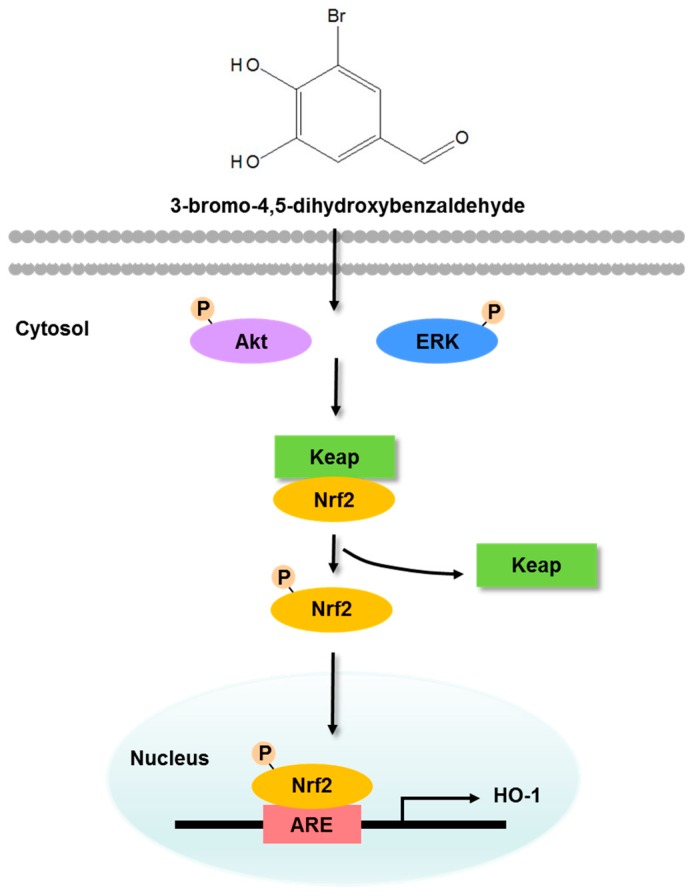
The compound 3-BDB protects human keratinocytes from oxidative stress by upregulating ERK and Akt, which allows Nrf2 to induce the transcription of the antioxidant enzyme HO-1.

Several studies have reported that ERK and Akt signaling pathways are involved in HO-1 expression and in Nrf2-dependent transcription in several cell types in response to oxidative stress. For instance, natural antioxidant compounds increase ERK and Akt phosphorylation, further inducing Nrf2 and HO-1 [28,30,34]. In the present study, ERK and Akt signaling induced HO-1 expression and Nrf2 phosphorylation. As shown in Figure 6, our results suggest that ERK and Akt regulate 3-BDB-induced Nrf2/HO-1 expression via ARE. Moreover, selective inhibitors of ERK and Akt pathways attenuated 3-BDB-induced HO-1 and phospho-Nrf2 levels by blocking ERK and Akt phosphorylation. These results suggest that Nrf2 downregulates ERK and Akt pathways. However, ERK and Akt are potentially important factors in the cellular signal transduction associated with the initiation of Nrf2 and transcriptional recruitment of HO-1 in keratinocytes. Further, a previous study has reported that 3-BDB potentially enhances antioxidants such as reduced glutathione [35].

The present results show that both ERK and Akt inhibitors significantly attenuate 3-BDB-induced phospho-Nrf2 and HO-1 by blocking ERK and Akt phosphorylation. This suggests that activation of several protein kinases, including ERK and Akt, results in Nrf2 phosphorylation and promotes the dissociation of Nrf2 from Keap1, thus activating the Nrf2 response to 3-BDB and Nrf2 accumulation in the nucleus. Furthermore, it has been reported that 3-BDB notably attenuated UVB-mediated intracellular ROS generation and cellular apoptosis [23]. 

Our results confirm that 3-BDB protects keratinocytes from damage due to oxidative stress. It exerts considerable antioxidant effects through the upregulation of ERK and Akt, allowing Nrf2 to induce the transcription of the antioxidant enzyme HO-1 (Figure 6). These findings provide novel insights into therapeutic approaches based on antioxidant compounds. The compound 3-BDB is suggested as a therapeutic candidate for skin aging, UVB-mediated skin damage, and inflammatory diseases of the skin.

## 4. Materials and Methods

### 4.1. Materials

The compounds 3-(4,5-dimethylthiazol-2-yl)-2,5-diphenyltetrazolium bromide (MTT) and ZnPP were purchased from Sigma Chemical Co. (St. Louis, MO, USA). Calbiochem (San Diego, CA, USA) provided U0126 and LY294002. Santa Cruz Biotechnology (Santa Cruz, CA, USA) supplied primary HO-1, β-actin, Nrf2, ERK, and phospho-ERK antibodies. Cell Signaling Technology (Beverly, MA, USA) supplied primary phospho-Akt (Ser 473) and Akt antibodies. Epitomics Inc. (Burlingame, CA, USA) provide the secondary rabbit-specific antibody. Other reagents were purchased as analytical grade.

### 4.2. Cell Culture

The human keratinocyte cell line HaCaT was purchased from Cell Lines Service (Heidelberg, Germany). Mouse MEFs from Nrf2 wild-type (Nrf2^+/+^) and Nrf2-null (Nrf2^−/−^) mice were provided by Professor Young Sam Keum (University of Dongguk, Seoul, Republic of Korea). Cells were cultured in Dulbecco’s Modified Eagle’s Medium (DMEM), with 10% FBS, streptomycin (100 µg/mL), and penicillin (100 units/mL) at 37 °C with 5% CO_2_ in a humidified atmosphere.

### 4.3. Reverse Transcription-Polymerase Chain Reaction (RT-PCR)

Cells (1 × 10^5^ cells/mL) were cultured in 100-mm dishes. After 16 h, the cells were treated with 3-BDB (10 µM, 20 µM, 30 µM, 40 µM, or 50 µM). At predetermined time points, total RNA was isolated with TRIzol^®^ reagent (GibcoBRL, Grand Island, NY, USA). The PCR program for HO-1 and the housekeeping gene glyceraldehyde 3-phosphate dehydrogenase (GAPDH) was as follows: 35 cycles of 94 °C for 45 s, 53 °C for 45 s, and 72 °C for 60 s. The primer pairs (Bionics, Seoul, Republic of Korea) were as follows (forward and reverse, respectively): HO-1, 5′-GAGAATGCTGAGTTCATG-3′ and 5′-ATGTTGAGCAGGAAGGC-3′; GAPDH, 5′-GTGGGCCGCCCTAGGCACCAGG-3′ and 5′-GGAGGAAGAGGATGCGGCAGTG-3′. The amplified products were resolved on 1% agarose gels, stained with ethidium bromide, and photographed under UV light [19].

### 4.4. HO-1 Activity

A previously described method was followed to assess HO-1 activity [24]. Briefly, we homogenized the cells in sucrose solution (0.25 M) with potassium phosphate buffer (pH 7.4, 50 mM) on ice. Then, we centrifuged the samples: 200× *g* for homogenates, 9000× *g* and 15,000× *g* sequentially for supernatants. The resulting pellet was suspended in potassium phosphate buffer (pH 7.4, 50 mM) and the total amount of protein in the suspension was quantified. The reaction mixture (200 µL) consisting of cell lysate (500 µg/mL), hemin (0.2 mM), rat liver cytosol (0.5 mg/mL), MgCl_2_ (0.2 mM), glucose-6-phosphate (2 mM), glucose-6-phosphate dehydrogenase (1 U/mL), NADPH (1 mM), and potassium phosphate buffer (pH 7.4, 50 mM) was incubated for 1 h at 37 °C. Chloroform (0.5 mL) was used to terminate the reaction. The absorbance of the chloroform layer at 464 nm and 530 nm was measured by a spectrophotometer to investigate the formation of bilirubin from the hemin substrate. 

### 4.5. Western Blotting

Cells were seeded in 60-mm culture dishes. After 16 h, the cells were cocultured with 3-BDB (30 µM). The cells were then harvested at the indicated time points. After washing with phosphate-buffered saline (PBS) twice, the cells were lysed by incubating in lysis buffer for 30 min. After centrifuging, the lysate was used to detect protein levels. The proteins were separated via SDS-PAGE in a 10% resolving gel. The separated proteins were transferred to nitrocellulose membranes (Bio-Rad, Hercules, CA, USA). Then, the membranes were incubated with primary and then secondary antibodies (Pierce, Rockland, IL, USA). Images of Western blots were obtained on a LAS-3000 (Fujifilm, Tokyo, Japan) using an enhanced chemiluminescence Western blotting detection kit (Amersham, Little Chalfont, Buckinghamshire, UK) [36].

### 4.6. Nuclear Extract Preparation

At predetermined time points, the cells were harvested. The harvested cells were lysed on ice using 1 mL lysis buffer (Tris-HCl (pH 7.9,10 mM), NaCl (10 mM), MgCl_2_ (3 mM), and N P-40 (1%)) for 4 min. The lysate was centrifuged, and the pellets were suspended in extraction buffer (20 mM N-2-hydroxyethylpiperazine-N′-2-ethanesulfonic acid (HEPES), pH 7.9), 20% glycerol, 1.5 mM MgCl_2_, 300 mM NaCl, 0.2 mM ethylenediaminetetraacetic acid (EDTA), 1 mM dithiothreitol (DTT), and 1 mM phenylmethylsulfonyl fluoride (PMSF)). The samples were maintained on ice and then centrifuged. The resulting supernatants were used to measure protein concentrations and were then stored at −70 °C [33] 

### 4.7. Immunocytochemistry

Initially, cells that had been seeded on coverslips were fixed with paraformaldehyde (4%). PBS containing 0.1% Triton X-100 was used to permeabilize the cells for 2.5 min. Then, the cells were incubated in PBS blocking medium for 1 h followed by anti-Nrf2 primary antibody for 2 h and fluorescein isothiocyanate (FITC)-conjugated secondary antibody (Jackson ImmunoResearch Laboratories, West Grove, PA, USA) for 1 h. The cells were stained with DAPI (Vector, Burlingame, CA, USA) and mounted on microscope slides. A Zeiss confocal microscope (Carl Zeiss, Oberkochen, Germany) and Zeiss LSM 510 software (Version 2.8, Carl Zeiss) were used to acquire the images [33].

### 4.8. Chromatin Immunoprecipitation (ChIP) Analysis

The Simple ChIP™ Enzymatic Chromatin IP kit (Cell Signaling Technology, Danvers, MA, USA) was used to conduct the ChIP assay. Cellular proteins were crosslinked with 1% formaldehyde and then digested with nuclease for 12 min at 37 °C. The digested chromatin was incubated with primary anti-Nrf2 antibody and normal rabbit IgG overnight at 4 °C with continuous shaking. ChIP-grade protein G magnetic beads were used to collect the immunoprecipitated complexes. The immunoprecipitate was eluted with ChIP elution buffer after washing the beads. The eluent was incubated at 65 °C for 30 min to reverse the crosslinking. Subsequently, proteinase K was added, and the reaction was incubated at 65 °C for 2 h. Spin columns were used to purify the immunoprecipitated DNA fragments. The DNA collected from the immunoprecipitated complexes was subjected to 35 cycles of PCR. The primers for the HO-1 gene promoter were as follows (forward and reverse, respectively): 5′-CCAGAAAGTGGGCATCAGCT-3′ and 5′-GTCACATTTATGCTCGGCGG-3′. The PCR products were resolved on 1% agarose gels, stained with ethidium bromide, and photographed under UV light to visualize the DNA bands [33].

### 4.9. MTT Assay

The MTT assay was performed to assess the cytotoxicity of 3-BDB by checking cell viability. The MTT assay detects viable cells by the reduction of tetrazolium salt by mitochondrial dehydrogenases [33]. Cells (3 × 10^5^ cells/well) were cultured in a 96-well plate and treated for 2 h with 30 µM 3-BDB, ZnPP (the inhibitor of HO-1,10 µM), U0126 (the inhibitor of ERK, 10 nM), and LY294002 (the inhibitor of Akt, 5 µM), with 1 mM H_2_O_2_ or 30 mJ/cm^2^ UVB for another 1 h. After 24 h, we added 2 mg/mL MTT stock solution per well in a total reaction volume of 250 µL. After 2.5 h, the formazan crystals in the cells were dissolved in dimethyl sulfoxide. The absorbance was measured at 540 nm using a scanning multi-well spectrophotometer [37].

### 4.10. Statistical Analysis

Data were represented as means ± standard error. Multiple-group comparisons were performed using analysis of variance, followed by Tukey’s post-hoc tests. A value of *p* < 0.05 was considered statistically significant.
